# Feasibility and effectiveness of thoracoscopic pulmonary segmentectomy for non-small cell lung cancer: Retraction

**DOI:** 10.1097/MD.0000000000028566

**Published:** 2022-01-14

The article, “Feasibility and effectiveness of thoracoscopic pulmonary segmentectomy for non-small cell lung cancer”,^[1]^ which appears in Volume 99, Issue 5 of *Medicine*, is being retracted. The authors contacted the Editorial Office requesting a retraction after two years of additional research and an increase in clinical cases found the results to be unreproducible. The authors also found errors in the grouping of cases and the statistical analysis. After careful consideration by authors and the Medicine Editorial Office, it was determined that the article must be retracted.
